# A Practical Treatise on the Diseases of Children

**Published:** 1847-01

**Authors:** 


Art. IX.—*A Practical Treatise on the Diseases of Children.* By James Coley, M.D., Member of the Royal College of Physicians in London, &c.; Author of a Treatise on the Remittent Fever of Infants, &c,; and of Essays on Phlegmonous Erysipelas, &c. 8vo. pp. 414. Philadelphia: Ed. Barrington & Geo. D. Haswell. 1846.

The catalogue of diseases treated of in this volume is a very formidable one; we have taken the pains to count the list, and find that it embraces two hundred and thirty-three affections, medical and surgical. As we have heretofore stated in the author's own words, it was the intention of Dr. Coley to give to the profession "a comprehensive work." He has studied to include in it all the maladies and casualties incident to infancy and childhood. Beginning with the diseases connected with the separation of the umbilical cord, he omits no affection, however trivial it may be, by which life in its first stages is endangered, or the comeliness of the person impaired. The minuteness of his subdivisions may appear to the reader, at first, to be carried to an unnecessary extent, and as being tedious and perplexing, but the young practitioner, making out his diagnosis at the bed-side, will not object to the work on this acconnt. Snch an one requires a guide, which will enable him to recognise all the forms of disease which show themselves on the skin, in the eyes, the eye-lids, the mouths, of children, many of which, the experience of every physician soon teaches him, are of a character greatly to embarrass the novice in his profession, We do not say that even with the aid of this volume, which enumerates so fully the disorders of children, the young practitioner, without the advantages of having seen disease itself, will always be able to attain a correct diagnosis. This, we presume, is hardly to be expected from books alone. Disease must be seen to be understood; but it is not in the power of all students to see a great variety of diseases before entering upon the practice, and to such as have not had access to hospitals, or. been much in the sick chamber, such works as this are almost indispensable.

The author is brief upon most of the points embraced in his volume, disposing, for example, of the diseases under his first head, in five pages, and of the affections of the eye in about five times as many. To imperforate anus he devotes something less than a page, remarking that, fortunately, it is a rare defect. The opening may be partially closed, or it may be "obstructed entirely by a membrane extending over the orifice, and projecting;" in either of which cases the procedure necessary is easy and successful, a slight operation removing the obstruction. But in other instances the rectum terminates at a distance of several inches from the situation in which the anus ought to be found; in some it terminates in the bladder or the vagina; and in others it is altogether wanting. We once saw a case in which the bowel had its termination in the vagina, and we made two unsuccessful attempts to extend it. The first operation was performed a few months after the birth of the child; the incision was slight. We undertook a second when the child was about a year old, and after it had begun to experience irritation and trouble from the passage of the hardening feces. This time we pushed the incision to the extent of two inches, but without finding the rectum. The child ultimately died, and we should not be disposed to enter upon the operation again.

Purulent ophthalmia is the earliest of the diseases with which the eyes of infants are affected. It commences, according to our author, about the third day after birth. As the inflammation progresses it is accompanied by extreme intolerance of light, and if not retarded by proper remedies the conjunctiva assumes a billious appearance, resembling inflammation in the mucous coat of the intestines.

The effects produced upon the cornea are various. There may be effusion of lymph between its superficial layers constituting temporary blindness; or ulceration of a destructive character may creep round its margin, and terminate in gangrene or mortification, and incurable opacity; during which process the lens may escape; or ulceration may present itself at one spot either through the mucous or serous envelope of the cornea, and terminate in protosis or staphyloma; or ramolissement or softening of the cornea may occur, followed by an aperture through which the humors of the eye may escape; or suppuration of the globe may be the final result. This softening of the cornea, it must be observed, however, is not peculiar to this disease, as it was produced by an experiment made by Magendie on a dog, which he had confined exclusively to the use of sugar for food, whereby it was reduced to extreme exhaustion and marasmus; and Dr. C. M. Billard has seen many infants in the Foundling Hospital at Paris afflicted with softening of the cornea and complete marasmus by long-continued gastro-intestinal disease, and had no palpebral inflammation. I may also add that softening of the cornea has appeared in my practice in the adult as well as in infants as a consequence of purulent ophthalmy, when the patient has been subject to derangement in the chylopoietic viscera, which in this disease as well as in iritis, it will be found essentially necessary to modify or remove. Mr. Middlemore has once seen dropsy of the eye follow from this form of purulent ophthalmy. As results of this disease the iris may become closed, the pupil may be rendered angular, the iris may form adhesions with the lens or cornea, or be projected through an ulcer in the cornea protosis of the iris.

It is well known that children born of women laboring under gonorrhœa are pretty sure of becoming affected with this ophthalmia, and the prevalent opinion has been, that the affection is produced in every case by acrid matters applied to the eye at the time of birth. The author combats this idea, and it appears to us with propriety.

This conjecture is not altogether supported by facts, as the disease has been known to occur and pass through all its stages and end in opaque cornea *in utero;* and congenital opacity has been observed to disappear gradually by absorption after birth. There can be no doubt that gonorrhœa and other acrid discharges are frequent causes; but, as far as my own observation has gone, I have been satisfied that the disease has originated from exposure to cold, and especially an easterly wind, more frequently than from any other cause; and my belief that such is the most common origin of the disease is confirmed by the extensive experience of Langenbeck of Vienna, who remarks that the children in the Foundling Hospital in that city who are exposed to cold, and nearly dead from starvation when admitted, are much more frequently attacked with purulent ophthalmy than those who have the advantage of maternal care in the Lying-in-Hospital, whose mothers are women of the lowest description and almost invariably laboring under gonorrhœa.

Hurtletoup attributes the frequency of purulent ophthalmy at the Foundling Hospital in Paris to the crowding together a great number of infants in badly ventilated wards, where, he may have added, they are left at that helpless age with only one or two attendants in each apartment. My own experience proves that the purulent ophthalmy of infants occurs during the month of May, when the easterly wind and erysipelas prevail in the proportion of 7 to 8 compared with all the other months in the year.

In the commencement of the disease the usual mild washes will generally be sufficient to effect a cure. One composed of sulphate of zinc and distilled water, in the proportion of two grains of the former to an ounce of the latter, may be employed. It is proper also to give the infant a few doses of carbonate of magnesia, and if fever attend, calomel in small doses should be administered. No other medicine acts so benignly on the systems of children as calomel. Sometimes the ophthalmia resists these measures, when the author recommends an injection of the nitrate of silver, in the proportion of from five to ten grains to the ounce of water.

The injection in every instance should be introduced frequently every day by means of a glass or bone syringe, passed under and along the upper eyelid at the outer angle. When the granular condition of the mucous membrane obstinately persists, the morbid surface should be rubbed daily with sulphate of copper; but it must be observed that such a state of that membrane would not be met with if local depletion be timely adopted. Hence, at the commencement of a severe attack, leeches should be applied to the upper eyelids; but after the acute stage has passed by, stimulants will be found the best applications. These opposite modes of treatment are rendered necessary by the state in which the disease is found when the practitioner is consulted, and therefore must not mislead the inexperienced into a belief that every case can be safely treated alone by stimulants. The nature of the disease is the same as that of purulent ophthalmy in adults, which it is well known cannot be safely treated, when severe, without active and decided sanguineous depletion in the commencement. When suppuration of the globe takes place, the patient experiences great suffering, which should be relieved by puncturing the cornea. Mr. Walker, of Manchester, is a strenuous advocate for the exclusively stimulant treatment. He says the best remedy, especially to prevent ulceration of the cornea, is the application of nitrate of silver in substance to the lining membrane of the palpebræ. His practice consists in applying the caustic not only to the inner surface of the lids, but to the chemosed, conjunctival covering of the front of the globe, and holding it there a few seconds. As the disease advances, he states that the facility of its application increases. He never uses bleeding of any kind, and repeats the caustic once or twice a-day, according to the state of the eye. When the ulceration has taken place he applies it twice a-day, and says little else is necessary.

Ulceration of the cornea is frequently the consequence of this inflammation of the eye, and, if not arrested, leads to a destitution of the organ. The author prescribes the following treatment:

While any considerable vascularity and extreme photophoby exist, it will be proper to apply leeches immediately below the lower eyelid, or to the temple. After the inflammation has been modified, the ulcer should be touched daily, or every second day, with nitrate of silver scraped to a point, and a little oil should be afterwards dropped between the eyelids. During chemosis from conjunctival inflammation, the vessels of the cornea are strangled, and Mr. Tyrrel has discovered that radiated incisions, dividing the cornea from the centre to the sclerotic, immediately stops the ulcerating and sloughing process. Care must be taken to let the incisions of the membrane pass hetween the recti muscles to avoid the large vessels. For the cure of ulceration of the cornea and incipient pannus, M. Ammon performs the following operation with a remarkably successful result; a transverse fold is made in the upper lid, and the base of this fold is pierced by a curved needle with two threads of cotton, which form a seton. The ends of this seton are then to be fixed on the forehead by means of a piece of diachylon, the eyelid being so sufficiently raised as not to touch the globe. This suspension of the eyelids has a double influence. It acts as a seton on one hand, and on the other preserves the eye from the contact of the inner surface of the eyelid, which is often granular, and produces and continues the inflammation. There is one objection to this operation, which is, that it excites erysipelas of the lids.

Some of the other affections of the eye treated of in this connexion, are opacity, and hernia and fistula of the cornea, pustular and scrofulous ophthalmia, and corneitis. Of the latter disease the author gives an extremely satisfactory account. It is one which is often neglected, but one in which the resources of our art are very great to repair the injury. We subjoin all that Dr. Coley says on the subject:

The cornea is composed of an outer mucous membrane, lamenæ, or layers, constituting its proper, horny structure, cellular membrane connecting these laminæ and a serous membrane, which secretes the aqueous humor. Acute inflammation commences with some intolerance of light and a cloudiness in the cornea, which are followed by a pink zone surrounding it, composed of minute blood vessels. At length a pain in the forehead, like neuralgia, comes on, and the cornea assumes a milk-white appearance. When the inflammation is confined to one portion of the cornea, there is a corresponding preponderance of the small pink vessels on its margin, as in some cases of iritis. Instead of the milky appearance, which is produced by the effusion of lymph between the laminæ, we sometimes find the cornea of a dark red color, approaching to purple, completely obscuring the iris, and becoming unnaturally prominent. This condition has appeared to me to proceed from the rupture of the congested blood vessels, and the extravasation of the blood globules destined to undergo the change, which takes place in the formation of pus. When the disease is more advanced, we find purulent matter in the place of the bloody appearance, which, when active means are employed, become absorbed; or it may burst externally, or into the anterior chamber, constituting hypopion. Inflammation of the cornea is sometimes partial, in which case some portion of the iris may be obscurely discovered. Children laboring under this disease are generally supposed to have incurable blindness, until the nature of the case is explained by some one conversant with ophthalmic surgery; for, although the inflammation is acute in the first instance, relief is seldom applied for by the poor and ignorant until temporary loss of sight has taken place. In some cases, on minute inspection, we perceive red vessels ramifying through the cornea, before vision is suspended by the deposit of lymph; and on viewing the surface of the cornea with a microscope we perceive numerous depression or cup-like cavities, or ulcers presenting a glassy appearance.

*Treatment.*—The most active measures are required to prevent suppuration, ulceration, softening or mortification. The patient should be well purged, and afterwards repeatedly cupped on the temple, and be put on a mercurial course, so as to keep up a decided action on the gums until the symptoms have disappeared. These cases are tedious, but there are none in the practice of ophthalmic surgery, which terminate in so satisfactory a manner when properly treated.

Case: November 1, 1837.—Miss G., aged ten years, was brought to me totally blind. I found the blindness had existed two months, and was occasioned by effusion of lymph within the posterior layers of the cornea, which quite obscured the pupil in each eye. The front layers of the cornea quite transparent. A slight vascularity of the cornea, but no zone was present. Leeches had in vain been repeatedly applied. I prescribed cupping on the temple, and a grain and a half of chloride of mercury once in four hours. On the 4th, a little sight was restored in the left eye. The front of the cornea projected particularly at the margin, as if the aqueous humor was too abundant. As the patient objected to the medicine in the form of powder, I prescribed four grains of pil. hydrargyri once in four hours. On the 6th, the sight in the right eye was improved, and the cornea projected in the same manner as that of the left eye. She was now directed to take ten grains of pil. hydrarg. twice a day. On the 7th the mouth became sore, and the sight improved in both eyes. From this time the lymph became rapidly absorbed, and by the repetition of pil. hydrarg. twice a day during a few weeks, the whole of the lymph disappeared, and the sight was perfectly restored.

Case: 1838.—William Head, aged twelve years, had acute inflammation in the cornea three months, accompanied with effusion of lymph, which obscured the iris and obstructed the rays of light; great intolerance of light, and a pink zone round the cornea. I directed the temple to be cupped, and the patient to take ten grains of pil. hydrarg. twice a day. On the 10th, the cupping was repeated, the pain of which he had complained being unrelieved. On the 24th, the intolerance of light was diminished, but the pain and zone round the cornea remained; the cupping was repeated; the mouth was sore. On the 1st July, the inflammation was nearly gone, and the sight almost restored; and in the course of another week every symptom of the disease disappeared.

Case: Nov. 16, 1841.—A young man, aged 18 years, had suffered with complete blindness five months, occasioned by corneitis. The effusion between the layers of the cornea was of a dark, purple color. He had intolerance of light and severe pain in the eyes. I prescribed ten grains of pil. hydrarg. twice a day. On the 26th, the mouth became sore; the front of the cornea was transparent, while the posterior part was completely obscured by a bloody effusion, which had completely interrupted the sight; the pain had entirely subsided. On the 5th of December, the lymph became straw-colored, and the sight was beginning to return; the mouth was very sore. On the 12th, the bloody and purulent appearance had left every portion of the cornea, and the intolerance of light had subsided; the pupil in one eye became now distinctly perceptible, and there was only a thin layer of lymph left in the cornea of each eye. On the 19th, the cornea became quite clear on one side of each eye, and before the end of the month all appearance of inflammation and opacity had ceased.

A variety of corneitis, of a scrofulous character, is mentioned by writers. All I need to observe here is, that when the disease occurs in scrofulous children it is generally secondary; the inflammation having migrated from some other structure of the eye. It is also distinguished from simple corneitis by an increased projection of the cornea, occasioned by a redundant secretion of the aqueous humor. With respect to treatment, it may require some modification. If the patient is weak and pallid, and if the mercurial treatment appears to disagree, the disulphate of quina may be substituted.

To diseases of the skin our author devotes seventy-four pages. He adheres to the classification of Willian, which he esteems more convenient than the *natural system* lately proposed by Wilson. The idea of founding these maladies on the anatomy and physiology of the skin is an ingenious one, but Dr. Coley shows that it has not been carried out.

Gangrene of the mouth was first described by a Dutch surgeon about the beginning of the seventeenth century. In Holland the disease is known by the name of *water-kanker*. The title of gangrene was given to it by Van Swieten. It has repeatedly prevailed as an epidemic in various countries, in which form we have more than once heard of it in this country. Our author quotes a writer, named Poussail, who affirms "that in the United States of America, 72 out of 240 infants were attacked with gangrene of the mouth after remittent or intermittent fevers." He does not mention where this statement is published, and we are not sure that the writer meant the remark as general. If so, it is not true. He may have witnessed the disease in an epidemic form, in which so large a proportion of children convalescing from fever suffered with gangrene of the mouth; but no such result is common, nor has it been so at any time in this country. Dr. Coley gives the following account of this rare affection:

This disease is almost peculiar to infants who are feeble at birth, or children whose health has been deteriorated by a moist and unwholesome atmosphere, imperfect nutrition, or some antecedent disease, as scarlet fever, measles, small-pox, &c. It first appears with a swelling on the cheek, the central part of which is much harder than the circumference, and marked by a dark red spot, the skin having an appearance like wax, or as if it were oiled. The swelling, which is seated in the cellular membrane, extends to and distends the upper eyelid, and resembles œdema. At this time the patient looks pale, his breath is offensive, he has but little fever, is unhappy, and seldom complains of any pain in the cheek or mouth. An ulceration forms in the middle of the cheek, on the inside of the mouth, or on the membrane covering the gum or lip, and is covered with a putrid, grey, fetid slough of a characteristic odor. This ulcer soon assumes a purple color, and the parts mortify. The child at one time is engaged with surrounding objects, at others, lies in bed indifferent to everything, without strength or energy. He appears bloated on one side of his face, and shrunk and miserable on the other; his bloody or blackish saliva flows through his mouth, and he is constantly asking for food. which he devours with avidity, together with the putrid sloughs separated from the gangrenous parts. His intellect is clear, except during the night, when he is occasionally delirious. Some time between the third and sixth day, the most hard and prominent part of the cheek assumes a purple color, and eschar forms, which is extended from day to day, so that it ultimately occupies, the whole of one side of the face, extending even to the neck. Meantime the mucous membrane is completely destroyed down to the bones. In this state the appearance of the child is hideous; his appetite continuing voracious, he is constantly snatching the gangrenous shreds into the mouth, while he allows the offensive and blackish sanies to flow upon him from all sides. After a while his appearance becomes still more repulsive, when the eschars are partly separated and expose an opening in the cheek, through which may be seen the bare and tottering teeth, and the jaw-bones black and denuded. The morbid mass is now still more putrid and repulsive, and the child, burning with thirst, drinks with avidity, and continues to take food voraciously, although quite exhausted. He has no sickness, but has generally toward the last a troublesome purging, accompanied with rapid emaciation. His pulse becomes small and insensible, and death supervenes without a struggle.

The remote cause of this rare disease appears to consist of a degraded condition of the general health, and particularly an enfeebled state of the capillary circulation, induced by some previous inflammatory or other exhausting disease, which has reduced the constitutional vigor. Of these antecedent diseases the most frequent is measles; the next in frequency is that cachectic state, which is found in the children of the poor, who are exposed to a humid and unhealthy atmosphere, and are deprived of propcr nutriment and clothing. The feeble state in which children are left by severe attacks of any other of the eruptive fevers, equally with the condition I have just mentioned, predisposes them to inflammatory diseases, by depriving the small vessels, which circulate so freely in the cellular membrane, of their natural contractile power. Hence, when the circulation in the cellular structure, interposed between the mucous and cutaneous membranes of the upper cheek or lip, is retarded by any cause, the vessels, having lost their proper contractile force, which is designed by nature to overcome accidental interruptions, enlarge and permit the blood to remain stagnant. In proportion to the resistance thus presented to the perfect circulation in the part affected, is the excitement or vis a tergo in the surrounding capillary arteries; an inordinate activity is instinctively commenced in their vessels, for the purpose of attempting to overcome the obstruction in the morbid part; and, as the transmission of their contents is impossible through the ramifications already deprived of their vitality, or incapable of obeying the natural stimulus of the blood, the serous part of that fluid is forced through their minute extremities into the adjoining cellular membrane, which, from its increasing infiltration, becomes distended and indurated, and presents on the out-stretched skin the pale, lymphatic appearance before described as partly characteristic of the disease. When the serous membranes covering the abdominal viscera, or the heart, &c., become the seat of vascular obstruction, and inflammation follows, the excited and distended vessels relieve themselves by discharging serum or coagulable lymph from the free surfaces, while the neighboring cellular structure, with which their adherent portion is connected, comparatively escape the force and pressure of the opposite arterial excitation. Until the process of infiltration within the lip or cheek is removed by early and proper treatment, all the small vessels near the original seat of the disease become successively strangled, as it were, by the increasing compression of the serum, which they have themselves effused; and thus death and putridity extend their destructive and offensive influence.

It will be interesting to know the most approved methods of treating gangrene of the mouth. The different modes are mentioned in the annexed quotation:

A disease so rapidly fatal as gangrene of the mouth, requires the most prompt and vigorous remedies, otherwise the patient will speedily fall into an incurable state. The utility and necessity of stimulating and escharotic applications will appear, from what I have said, to have been long ago obvious to all who have had much experience with this formidable malady. The principle on which they act so beneficially, appears to me to be that of stimulating into contraction the diluted vessels surrounding the dead parts, and still possessing vitality. The consequence of this contraction is the immediate cessation of that rapid process of inflammation, which, as I have explained before, is produced and extended by defect of tone or nervous energy in the capillary circulation. In slight cases it may be sufficient to rub off, with diluted hydrochloric acid, the muguet or ash-colored slough found on the mucous membrane; but when the black spot, apparent on the outside, which from the cessation of the circulation and the death of the part, begins to increase, a crucial incision should be made through it down to the living parts, and undiluted nitric or hydrochloric acid applied by means of lint fastened to a glass rod. After making the incision, Billard advises the application of butter of antimony, or the actual cautery, which was extolled by Bacon above all other remedies. Those who have witnessed the astonishing effect of undiluted nitric acid in arresting the destructive process of sloughing phagedæna, recommended by Mr. Welbank in a paper on the subject in the "Medico-Chirurgical Transactions," will have no hesitation in adopting this bold and decided practice, which has now, with different modifications, received the sanction of experience. Should the first application of the escharotic fail, it should be repeated daily, until the sloughing process has subsided. After each operation, the only other external remedies required will be at first an evaporating bread and water poultice, until the discharge has begun to subside, and the dead parts have exfoliated, and afterwards dossils of lint, or soft linen rag, moistened with warm water, which may be continued until the mutilated parts have been cicatrised.

Little reliance must be placed on internal remedies, notwithstanding it is the belief with some writers, particularly Siebert, that it is the result of a scorbutic diathesis. Should any symptoms of land scurvy have preceded the attack, it will be necessary, in addition to nourishing diet, to prescribe some preparation of bark, with some mineral and vegetable acid. It was the opinion of Dr. Underwood that the diarrhœa, which appears towards the termination of the disease, is occasioned by the offensive matter constantly swallowed by the patient. This symptom may be relieved by opium; and should the child recover, change of air may be found advisable.

The author has some exceedingly interesting remarks in relation to membranaceous sore throat, from which we make a few quotations. By the French the disease is termed *dyphthorite:*

It is confined to an inflammation of the pharynx, extending to the tonsils, which terminates in the formation of a false membrane. Aretæus was the earliest writer on the subject of angina gangrenosa, which he described under the titles of the Egyptian and Syriac ulcer. He divided the disease into two species, one in which the artificial membranes are small and white, and the other in which these productions were large, depressed, and exhaled a fetid smell, constituting the angina gangrenosa. About the year 1337, the latter disease appeared as an epidemic in Holland, and in other parts of Europe, and re-appeared in Spain at the commencement of the seventeenth century, and was described by Mercatus, Villareal, Nunnez, and other Spanish writes, as affecting the tonsils, extending into the air-passages, and producing death by suffocation, whence it received the name of *garotillo.* In 1818 the epidemic showed itself at Naples, where it became as fatal as in Spain, commencing its attack in the pharynx, producing dysphagia, and terminating its career by spreading to the larynx, and exciting suffocation, as if the patient had been strangled. Seventeen years after this period the disease reached Kingston, in North America, where it was more particularly confined to infants. From 1743 to 1748, this epidemic raged in Paris, according to the reports of it left us by Malonin and Chomel; and, a short time afterwards, Fothergill and Stow published accounts of an epidemic, malignant, sore throat, which prevailed in England, which was sometimes secondary, and in connexion with scarlatina, and at others primitive, and resembling, from the description of Stow, the disease under consideration. In 1671, Bard, of New York, published his sentiments on this subject, which partly corresponded with those of Bretonneau, who considered the disease to be analogous in its nature with that of croup, and the false membrane to be the product of a concretion. In proportion as the real nature of this disease has been unfolded by modern researches, it has been disarmed of much of its supposed malignity, and brought more under the control of art. It begins with inflammation of the mucous membrane of the soft palate, tonsils, and pharynx, terminating in the secretion of a false membrane, *without any ulceration or destruction of the true skin.* As the inflammation advances, it is apt to extend to the larynx, and to produce the symptoms and fatal results of croup. In one form of the disease, gangrene or sloughing of the inflamed parts take place, particularly in children of feeble constitution. The attack begins with a little fever, attended with a slight difficulty in swallowing. On inspecting the throat, the tonsils are perceived to be swollen, and small portions of white or yellowish lymph may be seen, resembling muguet, on different parts of the soft palate and pharynx. After a short time these deposits of lymph assume a grey color, and acquire an offensive odor; and a copious discharge of saliva flows from the corners of the mouth. At this period the cervical glands become inflamed and swollen. At length the grey lymph, constituting the false membrane, either falls off in a mass, and is ejected through the mouth, or it is separated in fragments and discharged by degrees, and is often reproduced. The appetite is little affected, and neither vomiting nor diarrhœa is present, unless the mucous coats of the stomach and bowels are the seats of the dyphtheritic production. When recovery commences, the false membranes cease to be reproduced, and the surface of the mucous membrane, by which they had been secreted, presents a red, excoriated aspect, without any degree of actual ulceration; the swelling in the cervical glands subsides; and, at the end of eight or ten days, recovery follows. In the more malignant cases, the disease extends into the air-passages, producing symptoms of laryngeal and tracheal inflammation. First hoarseness is observed; then a harsh, suffocating cough, accompanied with a croupy sound and an anxious expression, followed by a pale, cadaverous countenance, with the eyes sunk in their sockets; hurried and feeble pulse, cold skin; and terminating, when unrelieved, in irresistible stupor, a purple color of the lips, face, and extremities, and speedy death. When the bronchial tubes are visited by this disease, the cough becomes more frequent, the breathing more rapid, and accompanied with a mucous or rattling sound, and the patient sometimes expectorates shreds or tubular portions of lymph presenting a membranous appearance. This is frequently the sequel of laryngeal inflammation, and after the latter has been relieved, disappoints our hopes by a rapidly fatal termination.

Here, for want of room, we must close our notices of this excellent work.

